# Effects of an Alpha-4 Integrin Inhibitor on Restenosis in a New Porcine Model Combining Endothelial Denudation and Stent Placement

**DOI:** 10.1371/journal.pone.0014314

**Published:** 2010-12-13

**Authors:** Anne Braun, Lilibeth Dofiles, Serge Rousselle, Luis Guerrero, Jane Gunther, Ted Yednock, Alain Stricker-Krongrad, Elizabeth Messersmith

**Affiliations:** 1 Charles River Laboratories, Preclinical Services Massachusetts, Shrewsbury, Massachusetts, United States of America; 2 Elan Pharmaceuticals, Inc., South San Francisco, California, United States of America; 3 Alizee Pathology, LLC, Thurmont, Maryland, United States of America; Tufts University, United States of America

## Abstract

Restenosis remains the main complication of balloon angioplasty and/or stent implantation. Preclinical testing of new pharmacologic agents preventing restenosis largely rely on porcine models, where restenosis is assessed after endothelial abrasion of the arterial wall or stent implantation. We combined endothelial cell denudation and implantation of stents to develop a new clinically relevant porcine model of restenosis, and used this model to determine the effects of an α4 integrin inhibitor, ELN 457946, on restenosis. Balloon-angioplasty endothelial cell denudation and subsequent implantation of bare metal stents in the left anterior descending coronary, iliac, and left common carotid arteries was performed in domestic pigs, treated with vehicle or ELN 457946, once weekly via subcutaneous injections, for four weeks. After 1 month, histopathology and morphometric analyses of the arteries showed complete healing and robust, consistent restenotic response in stented arteries. Treatment with ELN 457946 resulted in a reduction in the neointimal response, with decreases in area percent stenosis between 12% in coronary arteries and 30% in peripheral vessels. This is the first description of a successful pig model combining endothelial cell denudation and bare metal stent implantation. This new double injury model may prove particularly useful to assess pharmacological effects of drug candidates on restenosis, in coronary and/or peripheral arteries. Furthermore, the ELN 457946 α4 integrin inhibitor, administered subcutaneously, reduced inflammation and restenosis in stented coronary and peripheral arteries in pigs, therefore representing a promising systemic therapeutic approach in reducing restenosis in patients undergoing angioplasty and/or stent implantation.

## Introduction

Coronary artery disease (CAD) is the leading cause of death in most developed countries. Percutaneous coronary intervention and implantation of stents have revolutionized the treatment of CAD. However, restenosis (narrowing of the vessel lumen) remains one major complication of coronary angioplasty and stent placement [1]. In this context, drug-eluting stents (DES; e.g. sirolimus- or paclitaxel-eluting stents) became the standard of care for the treatment of CAD, due to their strikingly reduced rates of restenosis compared with bare-metal stents [2], [3]. However, safety concerns with DES recently arose with reports of increased late in-stent thrombosis, possibly associated with increased mortality and myocardial infarction [4]. These findings have renewed interest for alternative or complementary interventions to reduce restenosis, including systemic pharmacological treatments after bare metal or drug-eluting stent placement.

Restenosis studies are performed to unravel the fundamentals of arterial healing to injury, or to assess preclinical safety or efficacy of novel devices or pharmacological treatments. They rely on a number of animal models, in which an initial injury to the arterial wall is applied [5]–[8], most frequently either by using angioplasty balloons to induce endothelial cell denudation, or by implanting stents. Cholesterol-raising diets have also been used to enhance lesion formation [9], [10]. Among these models, the pig is commonly used, because porcine heart and vasculature, anatomy and physiology, as well as pathophysiology of the neointimal response after vessel injury, present numerous similarities to those in humans [11]. Pig models have usually demonstrated good predictive value in subsequent clinical trials [4], [5], [8], [11], although current data shows better predictivity for safety than for efficacy assessments. For efficacy studies, one critical requirement of an appropriate model is consistent and sufficient neointimal hyperplasia, to subsequently allow valid assessment of the effects of the drug or device tested. This is particularly true for peripheral arteries, which show high elasticity and biomechanical compliance, and do not consistently exhibit sufficient neointimal hyperplasia.

*In vivo* studies show increasing evidence of the pivotal role of inflammation in post-stenting restenosis [12]. The inflammatory reaction resulting from arterial injury and accompanying the wound healing process influences neointimal growth and lumen narrowing [12], [13]. Inflammatory cells including monocytes, leukocytes, neutrophils and macrophages are recruited and accumulate as infiltrates primarily on the site of arterial wall injury [12]. In a rabbit arterial injury model, neutrophil accumulation on the medial and neointimal layer of the injured artery was reduced after administration of an anti-inflammatory agent [14]. Vascular cell adhesion molecule-1 (VCAM-1) is expressed on activated endothelium and mediates capture and migration of circulating mononuclear cells expressing α4β1 integrin [15]. Blockade of the VCAM-1 and α4β1 integrin (VLA-4) interaction was shown to decrease lymphocyte trafficking from the periphery into the vessel wall, attenuate leukocyte recruitment and neointimal formation [16]. In a rabbit carotid arterial electrical injury model, a 70% decrease in infiltrating neointimal mononuclear leukocytes was observed after administration of an anti-α4 integrin antibody [17]. In ApoE deficient mice, blocking the α4β1-VCAM-1 interaction using an antibody against α4β1 integrin resulted in reduction of monocyte rolling and adhesion after carotid air dessication injury [18], and in a 72% reduction in neointimal thickening [16]. In a porcine coronary artery model of single balloon injury, α4 inhibition was associated with significant decrease in neoadventitial formation (14 days post injury) and lumen narrowing (3 and 14 days after injury) [19].

ELN 457946 is a novel compound developed by Elan Pharmaceuticals, which inhibits the α4 integrin-mediated adhesion of leukocytes to VCAM-1. ELN 457946 has strong binding affinity to lymphocytes *in vitro* through α4 integrin, and subcutaneous administration of ELN 457946 *in vivo* resulted in increased lymphocytes counts in the blood (Dofiles and Gunther, Elan Pharmaceuticals; unpublished data; 2007). ELN 457946 was shown to resolve paralytic symptoms and to reverse histopathological changes of disease in animal models of multiple sclerosis (Dofiles, Elan Pharmaceuticals; unpublished data; 2006). Here, we use this compound to further explore the role of α4 integrin in arterial remodeling and restenosis.

Here we developed a double injury model, combining endothelial denudation with an angioplasty balloon and implantation of bare metal stents in normal swine arteries. One month after the interventional procedure, complete healing was observed in all animals, and area percent in-stent stenosis was 64% in the left anterior descending (LAD) coronary arteries, and approximately 15% and 30% in the iliac and carotid arteries, respectively. Therefore, this double injury porcine model leads to robust and consistent restenosis, even in highly elastic peripheral arteries, and may provide an improved preclinical model to observe pharmacological effects of candidate drugs on neointimal response. Using this model, we investigated the effects of ELN 457946, an α4 integrin inhibitor, on vascular healing and restenosis. We showed that ELN 457946-treated animals exhibited reduced inflammation in the stented vessels, as well as decreases in area percent in-stent stenosis of 12% in the LAD and 30% in the iliac and carotid arteries, compared to control animals.

## Methods

### Ethics statement

All animal care and procedures conformed with the Guide for the Care and Use of Laboratory Animals published by the US National Institutes of Health (NIH Publication No. 85-23, revised 1996) and were approved by Charles River Laboratories Institutional Animal Care and Use Committee.

### Animals, Treatments and Surgical procedure

Male Yorkshire pigs (Sus scrofa; 3 month; 28–32 kg; Animal Biotech Industries, Danboro, Pennsylvania) were maintained on standard pig chow. Animals were dosed subcutaneously with vehicle (phosphate buffered saline [PBS]; n = 6), or 30 mg/kg ELN 457946 (n = 7), once weekly for four weeks (Days -1, 7, 14, and 21). Animals received aspirin (81 mg, PO) and clopidogrel (75 mg, PO), starting 2 days before surgery and until necropsy; and nifedipine (30 mg, PO), cefotaxime (50 mg/kg, IV) and buprenorphine (0.01 mg/kg, IM), prior to surgery. Under isoflurane anesthesia, and after heparin administration (50–150 IU/kg, IV), an 8F arterial sheath (Medtronic) was inserted in the right common carotid artery. Under fluoroscopic guidance, a 6F guide catheter was advanced through the sheath to the LAD ostium. Endothelial cell denudation was performed using an angioplasty balloon catheter (NC Raptor PTCA, Cordis) advanced to the proposed location, appropriately inflated, and moved back and forth 5 times over approximately 1.0 cm. One stent (MicroDriver or Driver, 2.75–3.5 mm ×18 mm, Medtronic) was placed in the LAD, to achieve a target balloon-to-artery ratio of 1.1–1.2∶1. Endothelial injury (Sailor Plus, Admiral extreme PTCA balloon catheter, ev3) and stent deployment (Aurora, self-expandable nitinol stents, 6 mm ×40 mm, Medtronic) were similarly performed in one iliac and the left carotid artery. Angiographic images (OEC 9800® Fluoroscope and Visipaque™, GE Health Care) of the vessels were obtained on the day of surgery and necropsy. Euthanasia (sodium pentobarbital, 60 mg/kg IV and exsanguination) was performed on Day 30–31, in accordance with accepted AVMA guidelines.

### Clinical pathology and Pharmacokinetic analysis

Blood was collected from the precava in isoflurane-anesthetized animals. Hematology parameters were obtained at predose, 48 hours after each dose, and prior to necropsy (Bayer ADVIA 120 hematology analyzer; Siemens Diagnostics, Ramsey, Minnesota). Statistical analysis was performed using a Mann-Whitney test (significance at p<0.05). Plasma concentrations of ELN 457946 were determined, at predose and 0.5, 2, 4, 24, 36, and 48 hours after dose (first and fourth doses); at predose and 48 hours after dose (second and third doses); and prior to necropsy, using a research bioanalytical HPLC-MS/MS method. An internal standard was added and samples were prepared using protein precipitation and liquid-liquid extraction. Following chromatographic HPLC separation, ELN 457946 was quantified using tandem mass spectrometry. Analysis was performed using an API-3000 triple quadrupole mass spectrometer (Applied Biosystems, Foster City, CA) with electrospray ionization (ESI) in the positive ion mode. Pharmacokinetic analysis was conducted using WinNonlin (V5.2).

### Tissue processing, Histopathology and Quantitative Morphometry

The heart, descending aorta and brachiocephalic trunk were perfusion fixed with lactated Ringer's solution and 10% neutral buffered formalin. Stented vessels were dissected, embedded in epon (Spurr), trimmed at three levels (proximal, middle and distal), sectioned, and resulting slides were stained with hematoxylin and eosin and elastin trichrome. Neointimal hyperplasia was quantitatively assessed using histomorphometric measurements of the internal and external elastic lamina (IEL and EEL, respectively) and lumen area for each artery (Image-Pro® Plus software). Neointima area, media area, percent stenosis, lumen diameter, and neointima thickness values were derived from these measurements. Statistical analysis was performed using Microsoft® Excel Student's t-test (significance at p≤0.05). Semiquantitative analysis was performed using terminology and grading scales consistent with published recommendations (HSRL, 20 Frederick Road, Thurmont MD) to assess the biological response of vascular tissue to the stents [20]. A grading score of 0, 1, 2 or 3, corresponding to absent, slight or minimal, moderate, or marked biological response, respectively, and based on the extent of the circumference of the artery involved, was applied to each studied parameter. Main microscopic parameters analyzed included inflammation, neointima fibrin and fibrinoid deposits, neiontima maturity (degree of organization and presence of smooth muscle cells), media hypocellularity, adventitial fibrosis, endothelialization scores, and vessel wall injury.

## Results

### Study Design and Surgery

**Figure 1:**
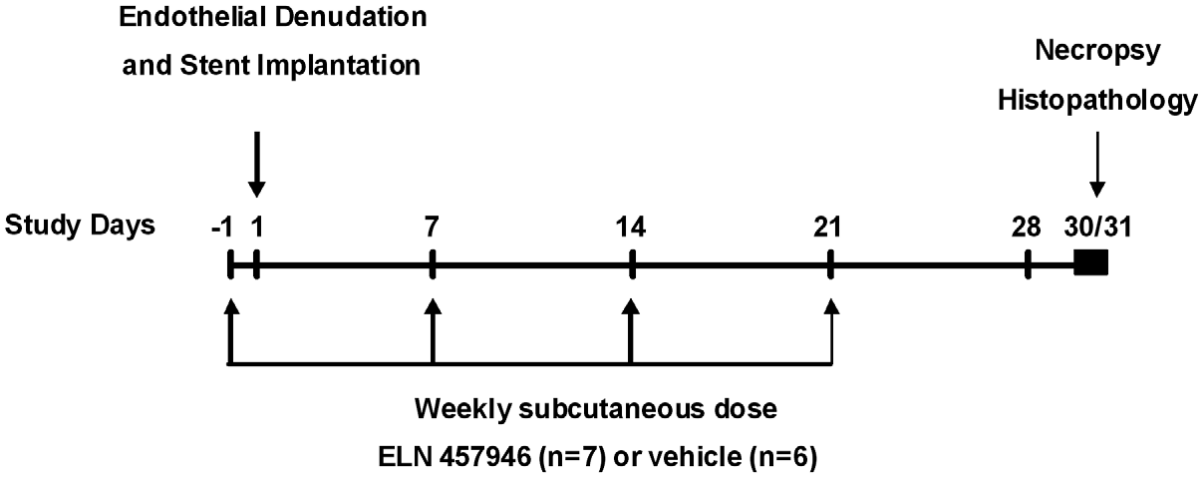
Study design.

A total of 13 male young adult Yorkshire pigs were dosed subcutaneously with vehicle (6 animals) or 30 mg/kg ELN 457946 (7 animals), once weekly, for a total of 4 injections (Figure 1). This dosing scheme was selected based on a previous pharmacokinetic study using a single 10 mg/kg subcutaneous dose in swine. The once weekly, 30 mg/kg dose of ELN 457946 was anticipated to provide appropriate exposure and efficacy throughout the study. The first dose was administered at least 24 hours prior to the surgical procedures. Animals underwent a single surgical procedure consisting of endothelial cell denudation by angioplasty balloon injury and subsequent implantation of one stent in each the left anterior descending coronary artery (LAD), a right or left iliac artery (RIA or LIA), and the left common carotid artery (LCCA), when possible. One month after surgery, the animals were euthanized and the stented vessels and adjacent areas were collected and prepared for histopathologic evaluation.

As a result of the surgical procedures, a total of 6 LAD, 6 iliac (5 RIA and 1 LIA), and 6 LCCA arterial stents were placed in 6 vehicle-treated animals, and a total of 6 LAD, 6 RIA, and 6 LCCA stents were placed in 7 ELN 457946-treated animals. Quantitative angiography confirmed that the desired stent:artery ratio of 1.1–1.2∶1 was achieved for all vessels in all animals, with the exception of the distal portion of the iliac artery in one ELN 457946-treated animal. Data from this vessel were excluded from all subsequent analyses.

No early deaths were observed after stent implantation. There were no abnormal clinical observations following treatment with ELN 457946 or vehicle. Animals in both treatment groups gained an average of 5.3–5.4 kg in body weight during the course of the study, and clinically tolerated well the surgical procedures and treatments.

### Double injury model: Histology of stented vessels

**Figure 2:**
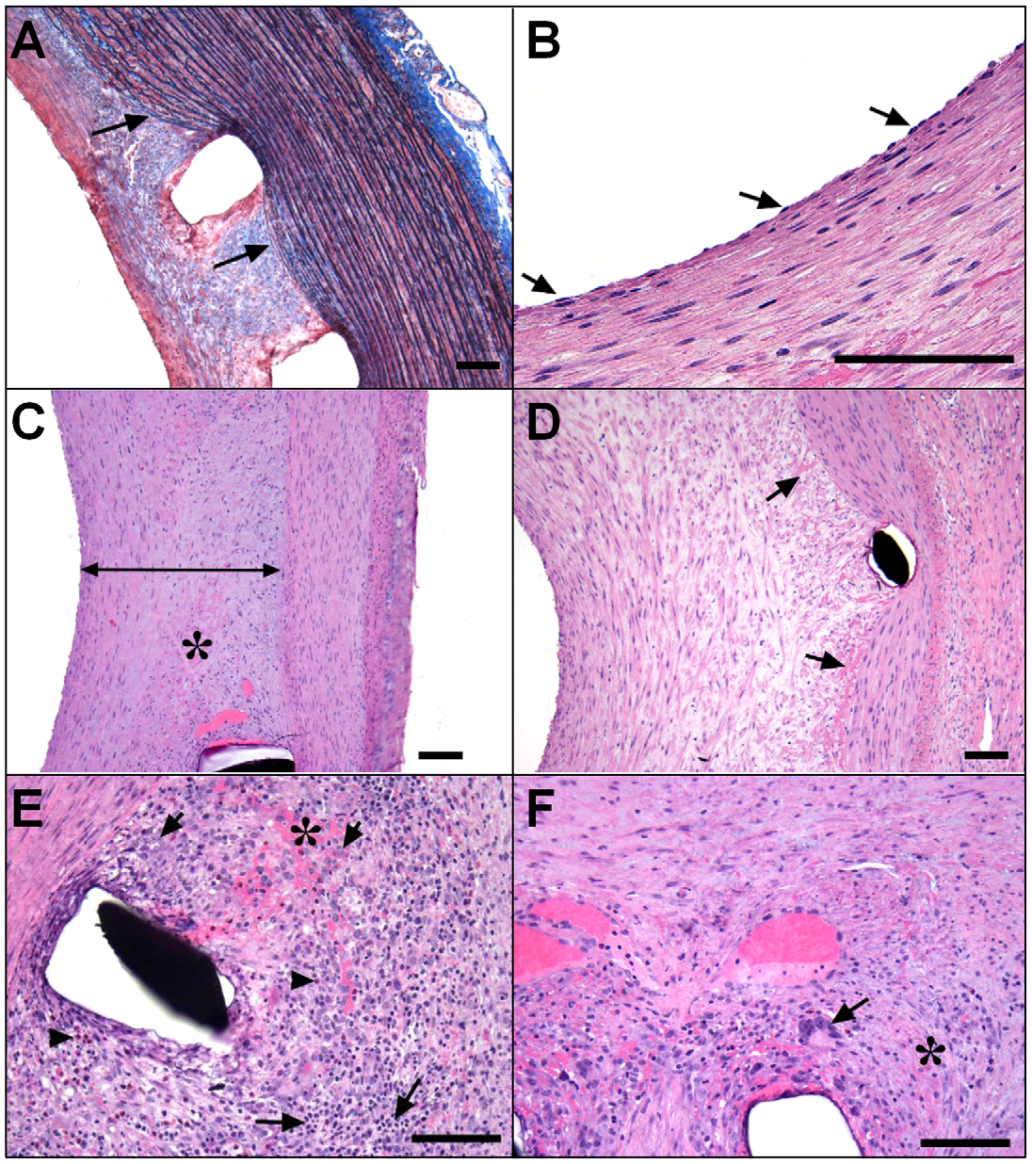
Histological analysis of the stented vessels. Representative images of elastin trichrome (A) and hematoxylin and eosin (B–F) stainings of sections in stented arteries. (A) Neointima injury (score 0). Arrows indicate intact elastic lamina in the carotid from a vehicle-treated animal. (B) Complete endothelialization in the LAD from a vehicle-treated animal. Arrows indicate endothelial cells. (C) Full thickness neointima with well-organized smooth muscle cells, in the RI artery from a vehicle-treated animal. (D) Neointima fibrin (score 1), as typically observed around the stent struts, in the LAD from a vehicle-treated animal. Asterisks in Panels C, E and F indicate areas of fibrin deposits. (E, F): Inflammation in the distal region of a right iliac artery from a vehicle-treated animal. (E) Arrowheads/long arrows/short arrows, indicate eosinophils, lymphocytes and macrophages, respectively. (F): Arrow indicates a foreign body giant cell. Bar = 100 µm.

Proximal and distal non-stented artery segments did not show sufficient neointimal response (data not shown) and were not evaluated further. Representative histology of stented vessels, harvested at euthanasia, one month after stent placement, is shown in Figure 2.

Procedural injury was nearly absent in the stented peripheral arteries, as reflected by intact elastic lamina and near lack of mural laceration (Figure 2A; average score of 0.00±0.00 for carotid, and 0.06±0.14 for iliac arteries), and was very minimal in the stented LAD (average score of 0.50±0.66). All individual vessels demonstrated successful stent implantation with no or very limited associated procedural injury, and were therefore suitable for further histopathology and histomorphometry analyses. Endothelialization of the neointima was complete in all stented LADs (Figure 2B; average score of 3.00±0.00), and most peripheral arteries (average score of 2.94±0.14 for carotid, and 2.72±0.44 for iliac arteries), independently of treatment group. Occasional incomplete endothelialization was observed for peripheral vessels, at levels where stent struts were not covered by neointima, and likely corresponded to procedural stent malapposition (underdeployment; data not shown). Optimal healing, with formation of a robust, stable full thickness mature neointima, with a well-organized and dense population of smooth muscle cells (Figure 2C; also see Figure 2A–D), was observed in all LAD (average score of 3.00±0.00) and peripheral vessels (average score of 2.94±0.14 for carotid, and 2.56±0.50 for iliac arteries). Occasional slightly incomplete maturation was observed in a few peripheral arteries, independently of treatment group (data not shown). Light dispersed deposits of fibrin/fibrinoid were observed around or in the area of the stent struts (Figure 2D and asterisks in Figure 2C, E–F) in stented LADs and peripheral arteries, in amounts which were consistent with the advancement of neointima organization, one month after bare metal stent implantation in pigs. In vehicle-treated animals, the inflammatory reaction observed was slight, and consisted of some scattered mononuclear inflammatory cells, mostly lymphocytes and macrophages (Figure 2E) and foreign body giant cells (Figure 2F), distributed around or in the area of the stent struts in LADs and peripheral arteries.

In conclusion, histological analysis of the stented vessels in this double injury pig model showed (i) the stent implantation generated no or very limited injury to the vessel wall; (ii) there was a robust neointimal response in both LADs and peripheral arteries, with typical features of the biological response of vascular tissue to the stents; therefore providing a solid preclinical model to study restenosis and observe subsequent pharmacological effects of drug candidates.

### Pharmacokinetics of ELN 457946

Plasma levels of ELN 457946 (α4 integrin inhibitor drug candidate) and pharmacokinetic parameters were evaluated as part of the restenosis study (4 weekly doses of 30 mg/kg). Area under the plasma concentration-time profile and maximal plasma concentrations increased over time, suggesting accumulation of ELN 457946 in the plasma with repeated weekly administration. By Week 4, mean peak plasma concentration (Cmax) was 865 µg/mL and time of maximal plasma concentration (Tmax) was 39.4 hours. Trough concentrations, obtained from blood samples collected 7 days post dose on Weeks 2, 3 and 4 suggested that steady-state levels had not yet been reached by Week 4 (concentration: 464 µg/mL), and were consistent with the slow elimination of this compound. In conclusion, all ELN 457946-treated animals were exposed to continuous systemic levels of ELN 457946 throughout the study, starting approximately 24 hours after the first dose until euthanasia, one month after treatment start.

### Effects of ELN 457946 on clinical pathology and inflammation

**Figure 3:**
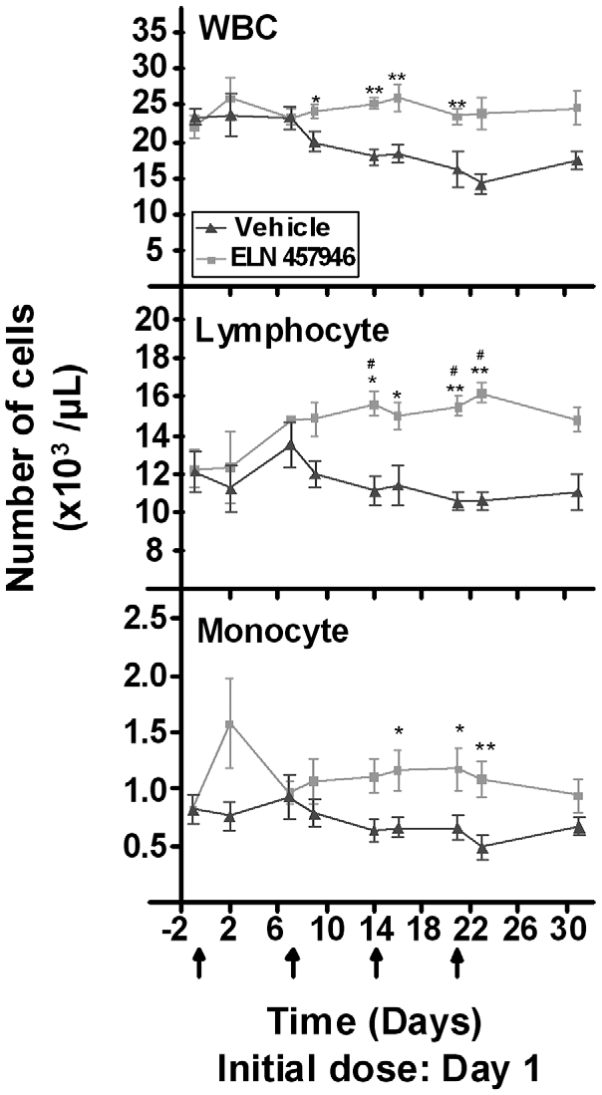
Effects of ELN 457946 on hematology parameters. Whole blood counts of white blood cells (WBC; top panel), lymphocytes (middle panel) and monocytes (bottom panel), over time after dosing with vehicle (black triangles) or ELN 457946 (grey squares). Dose administrations were done at weekly intervals (black arrows). Blood was collected prior to dose (predose or trough levels), 24 hours after the initial dose, and/or 48 hours post-dose, and prior to necropsy. * p<0.05 and ** p<0.01, when compared to vehicle group using Mann-Whitney. # p<0.05, when compared to baseline data (Day -1) using Mann-Whitney.

Hematology parameters, including white blood cells differential counts, were measured over the course of the study. All red blood cell parameters and eosinophil, basophil and reticulocyte counts were similar in ELN 457946- compared to vehicle-treated animals throughout the study (data not shown). Prior to the first ELN 457946 dose (Day -1, baseline), whole counts of white blood cells (WBC), lymphocytes and monocytes were similar in ELN 457946- and vehicle-treated animals (Figure 3). Starting 48 hours after the second dose (Day 9) and during the remainder of the study, mean WBC counts as well as lymphocyte and monocyte counts were consistently higher in ELN 457946-treated animals compared to vehicle-treated animals (Figure 3). Lymphocyte counts in ELN 457946-treated animals were also significantly increased compared to baseline lymphocyte counts. These changes in WBC, lymphocyte, and monocyte counts were consistent with the pharmacological activity of ELN 457946 and demonstrated that ELN 457946 was present and pharmacologically active in the blood circulation of ELN 457946-treated animals.

**Figure 4:**
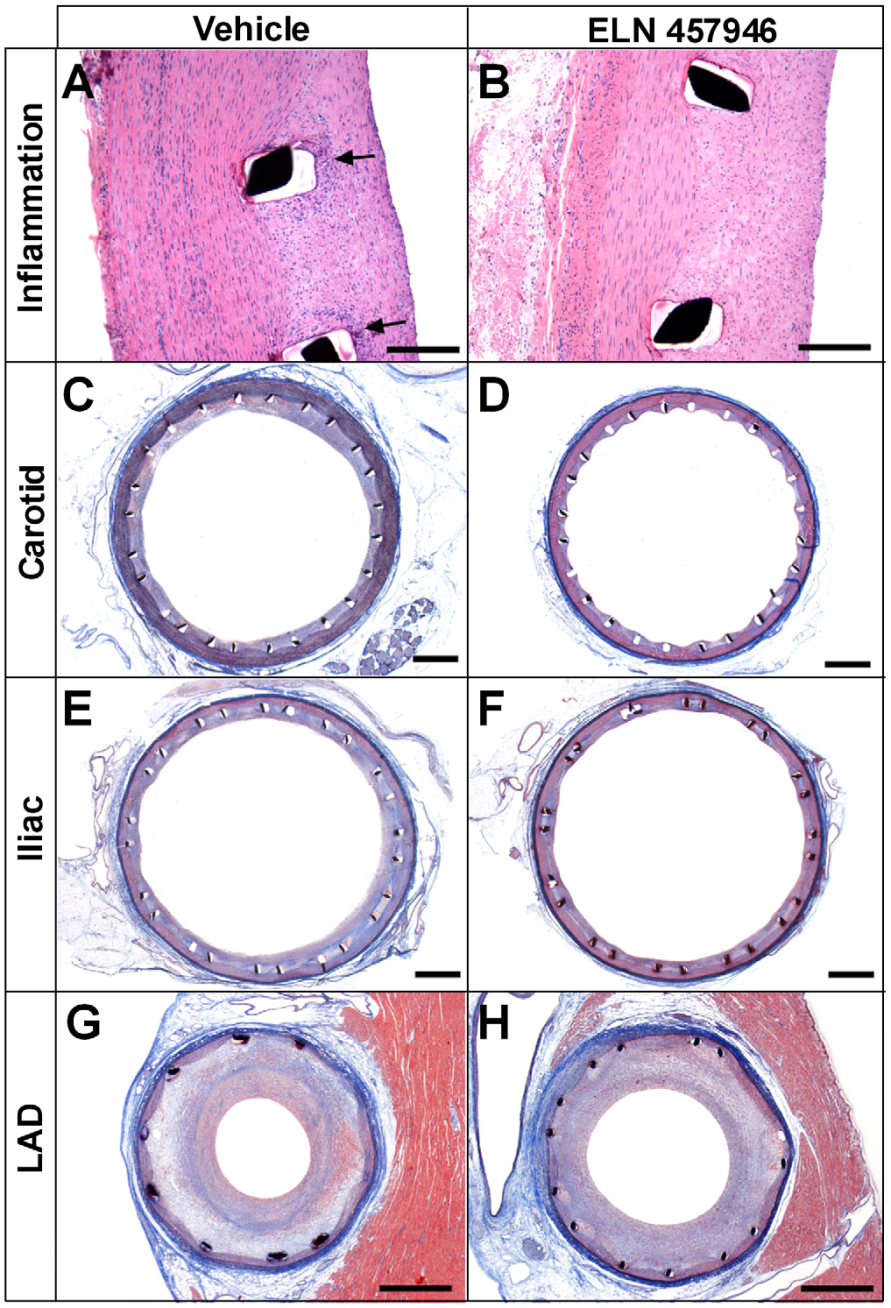
Effects of ELN 457946 on inflammation and restenosis. Representative hematoxylin and eosin (A, B) and elastin trichrome (C–H) stainings from vehicle- treated (left) and ELN 457946-treated (right) animals. (A, B): Inflammation in proximal region of stented left carotid artery. Arrows indicate inflammatory cells localized around the stent struts in vehicle-treated animal, absent in ELN 457946 animal. Bar = 200 µm. (C–H): Restenosis in stented left carotid (C, D), right iliac (E, F), and LAD (G, H) arteries. Bar = 1 mm.

Effects of ELN 457946 on inflammation were further investigated by qualitative and semi-quantitative histological analysis. While the overall inflammatory reaction was mild in LAD (average score of 0.33±0.56) and peripheral arteries (average score of 0.28±0.33 in carotid, and 0.78±0.66 in iliac arteries) from vehicle-treated animals (Figure 2E–F, and Figure 4A), there was virtually no inflammation in LAD and peripheral arteries from ELN 457946-treated animals (average score of 0.00±0.00 in LAD and carotid, and 0.07±0.15 in iliac arteries; Figure 4B). Vessel wall injury, a potent factor influencing inflammatory response to stents was low and comparable in control and treated groups. Therefore, treatment with ELN 457946 strikingly decreased the severity of the inflammatory reaction at the stent site in this double injury pig model.

In conclusion, four weekly treatments with 30 mg/kg ELN 457946 caused increased lymphocytes counts in the blood circulation (from decreased lymphocyte trafficking into inflammatory sites), and decreased inflammatory reaction to endothelial cell denudation and stent implantation at the stent sites in LAD and in peripheral arteries.

### Effects of ELN 457946 on restenosis

We then investigated potential pharmacological effects of ELN 457946 on restenosis in LAD and peripheral arteries. This double injury model produced average levels of restenosis around 60% in LAD, 30% in the iliac and ∼15% in the carotid arteries. The lack or very limited amount of procedural injury to the vessel wall allowed valid quantitative morphometry analyses for all stented vessels and comparison between treatment groups. For LAD, the average injury was slightly lower in the ELN 457946-treated group (score: 0.33±0.52) than in the vehicle-treated group (score: 0.50±0.66). However, the difference was small, within normal variability, and negligible with respect to the magnitude of the corresponding neointimal response. For peripheral vessels, the average injury was very low, and equivalent in the ELN 457946 and vehicle-treated groups (average scores between 0.00±0.00 in the carotid and 0.06±0.14 in the iliac).

Figure 4 shows vessel lumen and the extent of restenosis typically observed in carotid, iliac and LAD arteries (Figure 4C–D, E–F, and G–H, respectively). Quantitative morphometric measurements are summarized in Table 1. Peripheral (iliac and carotid) arteries from ELN 457946 and vehicle-treated animals exhibited similar average vessel size. After treatment with ELN 457946, neointima area and thickness were significantly decreased in carotid arteries and lumen area was significantly increased in iliac arteries. This resulted in approximately 33% and 29% statistically significant decreases in average percent stenosis in carotid and iliac arteries, respectively, following treatment with ELN 457946. Prenecropsy quantitative angiograms revealed changes in the arterial lumen sizes consistent with these findings (data not shown).

**Table 1:** Summary of histomorphometric measurements.

Artery	Treatment	Artery area(mm^2^)	Neointima area(mm^2^)	Lumen area(mm^2^)	Neointima thickness (µm)	Area stenosis(%)
LAD	vehicleELN 457946	7.68±1.458.27±1.45	4.10±0.593.91±0.94	2.51±1.363.16±1.38	581±127514±141	63.7±12.756.5±14.4
iliac	vehicleELN 457946	29.65±2.1530.39±0.95	7.51±2.785.48±0.64	18.62±2.8921.40±1.16 †	456±183318±43	28.8±10.220.5±2.8 *
carotid	vehicleELN 457946	28.00±1.4427.71±2.07	3.72±0.592.49±0.60 *	19.00±1.5420.21±1.29	231±43153±39 *	16.4±3.211.0±2.8 †

Data are means ± SD;

† *P*≤0.05;

*P≤0.01.

LAD arteries from ELN 457946-treated animals exhibited a slightly increased average vessel size compared to vehicle-treated animals (increased artery area; Table 1). Neointima area and thickness appeared slightly decreased and lumen area slightly increased, resulting in an approximately 12% decrease in average percent stenosis after treatment with ELN 457946. However, this decrease did not reach statistical significance. Since the magnitude of the decrease in stenosis was smaller in LAD than in peripheral arteries, and because of the slightly lower level of procedural injury observed in LAD arteries from ELN 457946-treated compared to vehicle-treated animals, the effects of ELN 457946 on stenosis in coronary arteries are less clear. However, the consistency of the variations observed across artery types, as well as the associated reduced inflammatory reaction, suggested the observed 12% decrease in LAD stenosis is related to treatment with ELN 457946.

## Discussion

In this study, we combined endothelial denudation by balloon angioplasty and bare metal stent placement in pigs, and characterized this new double injury model. A consistent low level of procedural injury was obtained, allowing valid comparison of the restenotic response between animals, a critical prerequisite for subsequent assessments. Complete healing was observed as evidenced by full or nearly full endothelialization, lack of substantial amounts of residual fibrin and complete maturation of the neointima into a stable fibromuscular layer covering all struts. The double injury procedure lead to a robust neointimal response in coronary arteries (average levels of restenosis around 60%), as well as in highly elastic, compliant- and stretch-resistant peripheral arteries (average levels of restenosis of 30% in the iliac and ∼15% in the carotid arteries). With the exception of the carotid arteries, these restenosis percentages correspond to a robust response, superior to what is typically observed one month after stenting alone in pigs. For example, percentage area stenoses of 42% (stent implantation only [9]), and 55% (stent implantation combined with a 13-week hypercholesterolemic diet and early endothelial abrasion [9]; or severe arterial injury with stents delivered on oversized angioplasty balloons [21]) were reported for porcine coronary arteries. In one previous report, combination of balloon injury using an oversized balloon and implantation of biliary stents led to a 44% area stenosis in swine femoral arteries, allowing to show significant differences in stenosis after intravascular sonotherapy [22]. In the context of preclinical efficacy studies, our double injury pig model appears particularly relevant to assess pharmacological effects of drug candidates on restenosis, including for peripheral arteries assessment. Future studies to evaluate and quantify effects of clinically efficacious drugs with different mechanisms of action, (e.g. rapamycin [12], [23] or tranilast [24], [25]) in this model may prove useful to further validate the model for coronary restenosis and to identify positive controls for preclinical assessments of new pharmacological agents. A number of different candidate drugs with various modes of action (for reviews, see [26], [27]), including anti-proliferative (e.g. cytarabine, doxorubicin, vincristine, colchicines, sirolimus), and anti-inflammatory agents (e.g. bisphosphonates, like clodronate, pamidronate, alendronate, ISA-13-1 [28], [29]); genes, growth factors and cytokines (e.g. PDGF receptor kinase inhibitors [30], or extracellular matrix metalloprotease MMP-9 [12]); lipids (e.g. ceramide [31]); and anti-platelet drugs (e.g. cilostazol [32]) are currently under investigation for the treatment of restenosis and could be tested in this double injury model.

Using this animal model, we tested an α4 integrin inhibitor, ELN 457946, and could demonstrate an important role for α4 integrin inhibition in the response to arterial injury. Pharmacological levels of ELN 457946 were achieved and maintained over the course of the study, with inflammatory cell infiltration in LAD and peripheral arteries fully inhibited. A small reduction (∼12%) in area percent stenosis was observed in LAD arteries, and was associated with reduced inflammatory response. This decrease did not reach statistical significance but was consistent with effects on other morphometric measurements, suggesting this decrease in LAD neointimal response was the result of treatment with ELN 457946. There was a statistically significant decrease (∼30%) in area percent stenosis in treated peripheral arteries when compared to the controls, with a clear relationship to ELN 457946 treatment.

The response to ELN 457946 treatment appeared quantitatively more important in peripheral arteries than in coronary arteries. This might be explained in part by the more important restenosis response, classically observed in the coronary arteries compared to more elastic peripheral vessels [33]. This apparent difference was not considered to reflect a different response to treatment, since all vessels (LAD, carotid and iliac) exhibited qualitatively similar healing and inflammation characteristics after treatment with ELN 457946.

Further refinement of the dosing scheme of ELN 457946 and of timing in relation with stent placement (e.g. second dose administered 1 week before stent placement, to achieve higher blood levels of ELN 457946 before surgery) might allow to observe more significant differences in the coronary restenosis response.

Our results are particularly interesting in light of recent controversies over safety concerns with the use of drug-eluting stents (DES). Drug-eluting stents (DES) have shown remarkable effectiveness in reducing neointima formation and in-stent restenosis [8]. However, incidences of acute, subacute or late thrombosis events have been reported recently with use of DES, due to incomplete healing (absent endothelialization, increased level of inflammation and unresorbed fibrin deposits) [4]. Recently, comparison of clinical outcomes in patients treated with either bare metal stents or drug-eluting stents has revealed similar 1- year mortality, myocardial infarction, and a trend toward increased target-vessel revascularization in DES vs bare metal stented arteries [34], [35]. In addition, chronic inflammatory reactions to polymer coating and the potential for hypersensitivity reactions is also a long term concern for current drug eluting stents [36], [37]. These findings have deeply renewed the interest of the scientific and medical communities, for alternative or complementary interventions to reduce restenosis, including systemic pharmacological treatments in conjunction with bare metal or drug eluting stent placement. Based on our results, ELN 457946 represents a promising therapeutic approach in this context, and further investigation should determine the clinical relevance of the effects observed in this new pig model.

## Acknowledgments

The authors wish to acknowledge Mark Dreyer and Linda Chen for PK analysis, and George Tonn, John-Michael Sauer, Nancy G. Wehner, Vince Mendenhall and the Charles River surgery technician's team for scientific guidance and/or technical support.
